# P-1391. Epidemiological Characteristics and Clinical Manifestations of Mpox among People with and without Vaccination: An Institutional Experience

**DOI:** 10.1093/ofid/ofae631.1567

**Published:** 2025-01-29

**Authors:** Minji Kim, En-Ling Wu, Yingbao Wang, David Aaby, Ying Cheung, Karen M Krueger, Maureen K Bolon, Shannon Galvin

**Affiliations:** Northwestern University, Chicago, Illinois; University of Chicago, Chicago, Illinois; Northwestern University, Chicago, Illinois; Division of Biostatistics, Department of Preventive Medicine, Northwestern University, Chicago, Illinois., Chicago, Illinois; Division of Biostatistics, Department of Preventive Medicine, Northwestern University, Chicago, Illinois., Chicago, Illinois; Northwestern University Feinberg School of Medicine, Chicago, Illinois; Northwestern University Feinberg School of Medicine, Chicago, Illinois; Northwestern University Feinberg School of Medicine, Chicago, Illinois

## Abstract

**Background:**

Since May 2022, there have been over 90,000 cases of mpox, with over 32,000 cases in the US. The smallpox and mpox vaccine modified vaccinia Ankara-Bavarian Nordic (MVA-BN; JYNNEOS), which was approved by the FDA in 2019, is currently the predominant vaccine used to prevent mpox infections. Data on vaccine efficacy varies, ranging from 66% to 86-88% with complete vaccination. This study aims to delineate the epidemiological characteristics, as well as the clinical features and outcomes in those who were unvaccinated and vaccinated in one institution.

**Figure:**
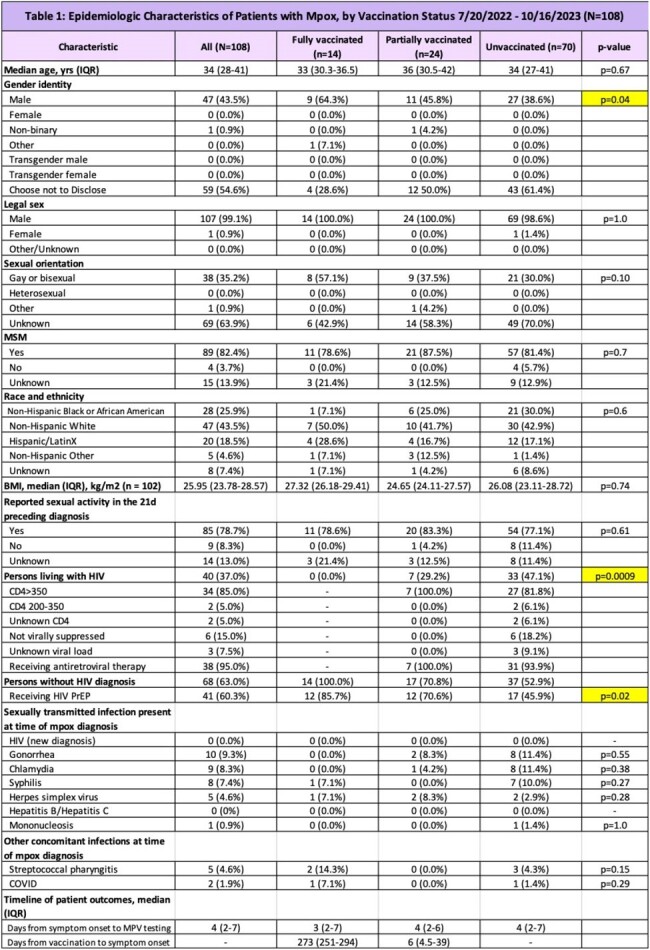

**Methods:**

This is a retrospective analysis of data obtained via EHR chart review and managed Research Electronic Data Capture tools with approval by the Northwestern University Institutional Review Board. Key descriptive characteristics included baseline demographics, clinical manifestations, concomitantly diagnosed sexually transmitted infections, and clinical outcomes (including markers of disease severity such as the mpox severity score system, Mpox-SSS). Vaccinated, partially vaccinated, and unvaccinated groups were compared using Fisher’s exact tests (categorical characteristics) and one-way ANOVA (continuous characteristics).

**Figure:**
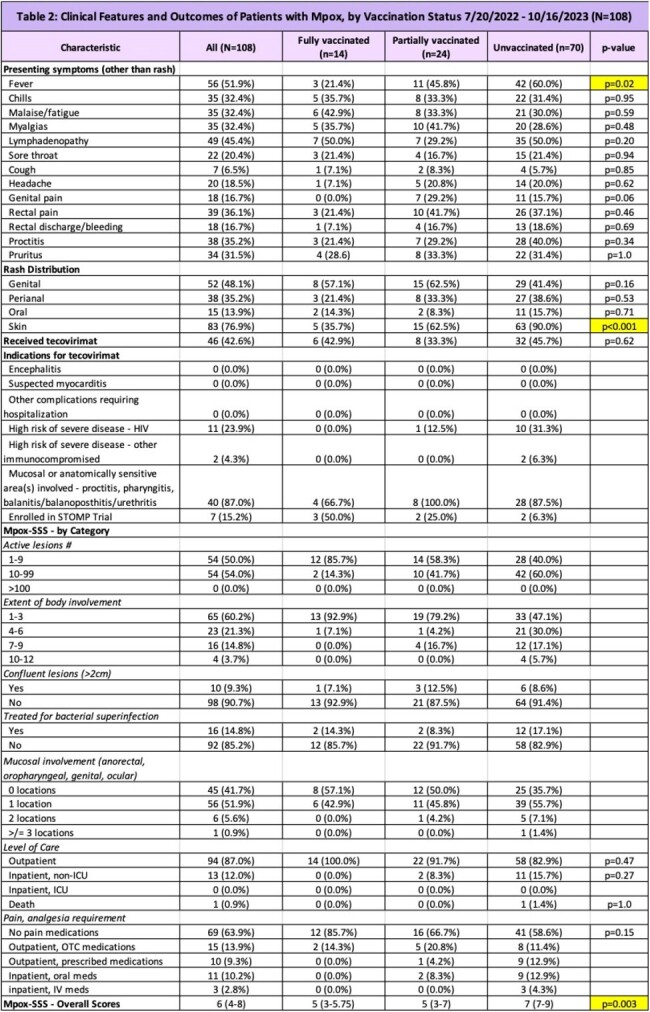

**Results:**

There were 108 individuals with PCR-confirmed mpox at our institution between July 20, 2022 and October 16, 2023. Of these 108 individuals, 14 were fully vaccinated, 24 were partially vaccinated, and 70 were unvaccinated. Baseline demographics are reported in Table 1. Unvaccinated persons differed from vaccinated and partially vaccinated persons in HIV status and with respect to PrEP usage. Clinical outcomes are reported in Table 2. Notably, median Mpox-SSS scores were as follows: 5 in the fully vaccinated group, 5 in the partially vaccinated group, and 7 in the unvaccinated group (p=0.03). There were no hospitalizations, ICU stays, and deaths in the fully vaccinated group but 11 hospitalizations (p=0.27) and 1 death in the unvaccinated group.

**Conclusion:**

Epidemiologically, both the vaccinated and unvaccinated population consisted predominantly of cisgender men who have sex with men (MSM). There were differences in HIV status and PrEP usage. Overall, compared to the unvaccinated group, the vaccinated group appears to have clinical features and outcomes that were less severe.

**Disclosures:**

**All Authors**: No reported disclosures

